# Decoding Physical and Cognitive Impacts of Particulate Matter Concentrations at Ultra-Fine Scales

**DOI:** 10.3390/s22114240

**Published:** 2022-06-02

**Authors:** Shawhin Talebi, David J. Lary, Lakitha O. H. Wijeratne, Bharana Fernando, Tatiana Lary, Matthew Lary, John Sadler, Arjun Sridhar, John Waczak, Adam Aker, Yichao Zhang

**Affiliations:** Hanson Center for Space Sciences, University of Texas at Dallas, Richardson, TX 75080, USA; david.lary@utdallas.edu (D.J.L.); lhw150030@utdallas.edu (L.O.H.W.); ashen.fernando@utdallas.edu (B.F.); tatiana.lary@utdallas.edu (T.L.); matthew.lary@utdallas.edu (M.L.); jcs170001@utdallas.edu (J.S.); arjun.sridhar@utdallas.edu (A.S.); john.waczak@utdallas.edu (J.W.); adam.r.a1@utdallas.edu (A.A.); yxz154930@utdallas.edu (Y.Z.)

**Keywords:** holistic sensing, particulate matter, physiology, machine learning

## Abstract

The human body is an incredible and complex sensing system. Environmental factors trigger a wide range of automatic neurophysiological responses. Biometric sensors can capture these responses in real time, providing clues about the underlying biophysical mechanisms. In this prototype study, we demonstrate an experimental paradigm to holistically capture and evaluate the interactions between an environmental context and physiological markers of an individual operating that environment. A cyclist equipped with a biometric sensing suite is followed by an environmental survey vehicle during outdoor bike rides. The interactions between environment and physiology are then evaluated though the development of empirical machine learning models, which estimate particulate matter concentrations from biometric variables alone. Here, we show biometric variables can be used to accurately estimate particulate matter concentrations at ultra-fine spatial scales with high fidelity (r${}^{2}$ = 0.91) and that smaller particles are better estimated than larger ones. Inferring environmental conditions solely from biometric measurements allows us to disentangle key interactions between the environment and the body. This work sets the stage for future investigations of these interactions for a larger number of factors, e.g., black carbon, CO_2_, NO/NO_2_/NO_*x*_, and ozone. By tapping into our body’s ‘built-in’ sensing abilities, we can gain insights into how our environment influences our physical health and cognitive performance.

## 1. Introduction

Over four million premature deaths worldwide were attributed to outdoor air pollution in 2016 [1]. In 2019, 99% of the global population resided in areas that fell short of the World Health Organization (WHO) air quality guidelines [1]. There is mounting evidence that poor air quality negatively impacts respiratory, cardiovascular, and cerebrovascular health [2,3,4,5,6,7]. Further, there is emerging evidence on the impact of poor air quality on neurological outcomes including chronic diseases (e.g., Alzheimer’s disease and dementia) [2,8,9] and acute cognitive impairment [10,11,12,13,14].

Although several large-scale epidemiological studies show the negative effects of air pollution on physical and cognitive health [2,3,4,5,6,7], these studies largely focused on coarse spatial (∼10 miles) and temporal (∼1 day) scales. Much less research focuses on ultra-fine spatial (∼1 m) and temporal (∼10 s) scales that make simultaneous environmental and holistic biometric observations of the human physiological responses.

Before an extreme result such as a disease occurs, poor air quality already negatively impacts human physical and cognitive performance [10,11,12,13,14]. Through this work, we investigate how air pollution impacts human health and performance by examining the relationship between environmental air quality measurements and automatic physiological responses at ultra-fine scales. Additionally, this work establishes the groundwork for future investigations by developing an experimental paradigm based on two main ingredients: holistic sensing and machine learning. Holistic sensing aims to capture all the relevant information about a system of interest. Machine learning is a framework that allows computers to learn by example and enables the development of high-fidelity empirical models [15].

This pilot study extends past works that examined interactions of cardiovascular variables such as heart rate (HR), heart rate variability (HRV), and blood pressure (BP) with air quality on fine scales [16,17,18]. The main contribution of this prototype study is that we augment cardiovascular markers with other biometrics, including electroencephalography (EEG), pupillometry, galvanic skin response (GSR), body temperature, oxygen saturation (SpO${}_{2}$), and respiration rate (RR). This extended set of variables captures both the cardiovascular and cognitive status of the participant. A study of air quality and human physiology at the ultra-fine level may shed light on the biophysical mechanisms that underlie their interactions.

## 2. Materials and Methods

### 2.1. Holistic Sensing

**Figure 1:**
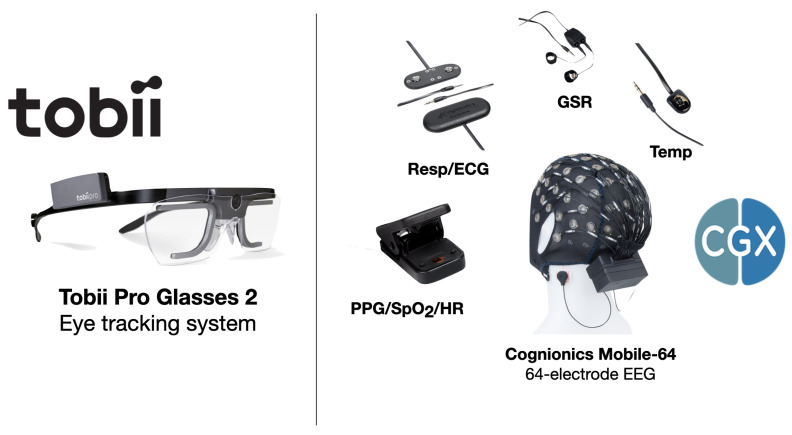
Biometric sensing systems. (Left) Tobii Pro Glasses 2 eye tracking system. This instrument performs eye tracking data, pupillometry, and provides two videos streams of the participant’s POV and eyes, respectively; (right) Cognionics Mobile-64 and AIM2 systems. Sensing suite includes 64-electrode EEG, PPG which measures SpO${}_{2}$ and HR, respiration/ECG sensors, GSR, and temperature probe.

**Figure 2:**
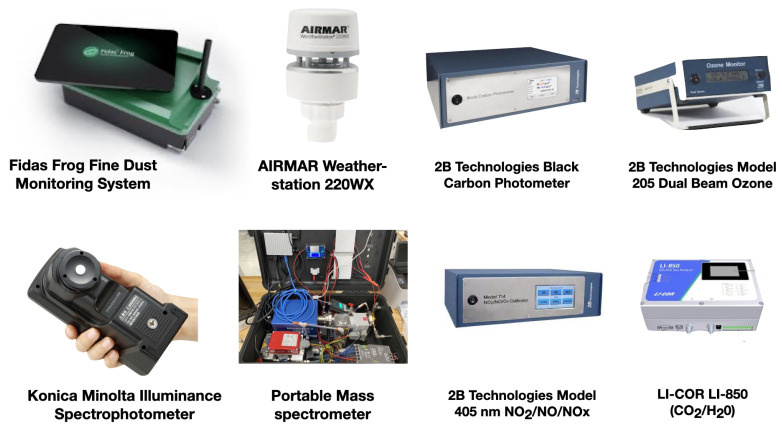
Images of full environmental sensing suite. Fidas® Frog Fine Dust Monitoring System measures particulate matter concentrations at 100 different size bins. The AIRMAR 220WX WeatherStation® Instrument samples barometric pressure, wind speed and direction, ambient temperature, and more. The 2B Technologies Black Carbon Photometer measures atmospheric black carbon particulates using long-path photometry. The 2B Technologies Model 205 Dual Beam Ozone sensor is a UV-based ozone monitor. The Konica Minolta CL-500A Illuminance Spectrometer measures the spectral irradiance from 360 to 780 nm at every nanometer. The portable mass spectrometer was constructed by the UNT Laboratory of Imaging Mass Spectrometry and measures charge mass ratios ranging 1–300 amu. The 2B Technologies Model 405 nm NO${}_{2}$/NO/NO${}_{x}$ Monitor™ directly measures atmospheric Nitrogen Dioxide (NO${}_{2}$) and Nitric Oxide (NO). The LI-COR LI-850 Gas Analyzer measured CO${}_{2}$ and water vapor in the air.

The data in this pilot study are a subset of a holistic biometric and environmental sensing paradigm. The goal of holistic sensing is to capture all relevant information about a system of interest. The full sensor array includes biometric monitors such as electroencephalography (EEG), eye tracking glasses, electrocardiography (ECG), galvanic skin response (GSR), body temperature, blood oxygen saturation (SpO${}_{2}$), and heart rate (HR), in addition to environmental factors such as particulate matter (PM), chemical composition of air, temperature, pressure, humidity, visible light spectrum, and more. The full array of biometric and environmental sensing systems are shown in Figure 1 and Figure 2, respectively. After processing raw sensor recordings, this full sensor array has a feature space approaching 20,000 variables (∼16,500 biometric and ∼2000 environmental). In the present study, we focus on a relatively small subset, consisting of 329 biometric and 51 environmental variables.

**Figure 3:**
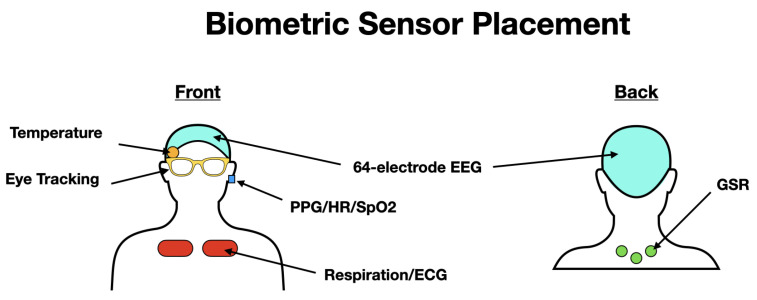
Schematic of biometric sensor placement on participant. (Left) Cartoon of front participant view. The 64-electrode EEG sits on the participant’s head. A temperature probe is placed under the EEG cap on the right temple. Eye tracking glasses are carefully placed on participant, avoiding EEG electrodes. PPG sensor is secured to left ear lobe. Respiration sensors are place near the top of the chest. (Right) Cartoon of back participant view. GSR sensors are placed below the back of the neck.

**Figure 4:**
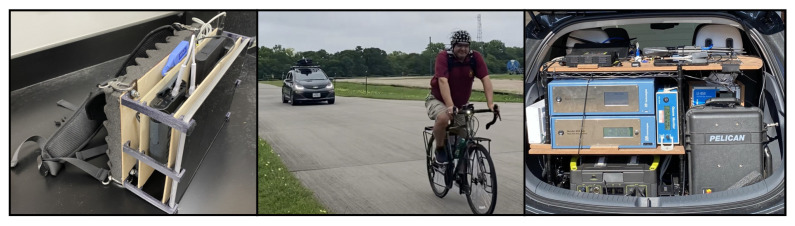
Data collection images. (Left) custom-made backpack to house biometric devices and recording computer; (middle) participant and environmental survey vehicle riding in tandem during data collection; (right) environmental sensors organized in trunk of electric survey vehicle.

The biometric sensing suite used in this research aims to comprehensively capture the physiological and cognitive status of the participant without restricting the participant’s actions, movements, or decision-making. The goal is to gather the maximum amount of information with minimal interruption of normal behaviors. Biometric sensors are placed on the participant in such a way to allow for unrestricted mobility (Figure 3). Sensor recording units and other devices are organized in a backpack worn by the participant that all together weighs less than 10 lbs (left panel in Figure 4).

Over 100 biometric markers are measured at sampling rates of 500 Hz and 100 Hz. These quantities are processed to derive over 329 variables for the present analysis. This holistic biometric sensing suite integrates two independent sensing systems which are shown in Figure 1. Eye tracking is recorded 100 times a second using the Tobii Pro Glasses 2. Data from the glasses produced average pupil diameter, the difference in pupil diameter between left and right eyes, and the 3D spatial distance between pupil centers. All other biometric data are measured 500 times a second using the Cognionics Mobile-64 and AIM2 systems. These systems include a 64-electrode EEG, temperature sensor, respiration sensor, photoplethysmogram (PPG), and galvanic skin response (GSR) measurement. Heart rate and SpO${}_{2}$ values are automatically computed by the AIM2 system using the PPG. Heart rate variability (HRV) and respiration rate (RR) are derived from respiration sensor data with a custom MATLAB script. All biometric data were down-sampled to 1/30 Hz (every 30 s) to match particulate matter recordings.

A holistic evaluation of an environmental setting is the ultimate goal of the sensing suite used in this study. This suite brings together several sensing packages, including fine dust monitoring from the Fidas® Frog, temperature, humidity, pressure, and wind speed and direction recorded with the AIRMAR Weatherstation 220WX; the full spectrum of visible light (360–780 nm) captured by the Konica Minolta Illuminance Spectrophotometer; dedicated gas monitors for black carbon, ozone, NO/NO${}_{2}$/NO${}_{x}$, and CO${}_{2}$/H${}_{2}$O; as well as a portable mass spectrometer (Figure 2). However, due to its significant societal relevance, for this pilot study, we focus on particulate matter (PM) concentrations recorded using the Fidas® Frog fine dust monitoring system. This instrument simultaneously measures PM mass fractions of PM${}_{1}$, PM${}_{2.5}$, PM${}_{4}$, PM${}_{10}$, and a distribution within a size range of 0.18–100 micrometers, as well as the total particle count density (dCn). PM data were recorded at sampling rate of 1 Hz and down-sampled to 1/30 Hz (every 30 s).

### 2.2. Data Collection

Biometric data collection was restricted to a single participant due to logistical constraints arising from the COVID-19 pandemic. However, future works will include data from multiple participants. The small population size in the present study is mitigated by two factors. First, data were collected over three separate days, providing a range of contexts. Additionally, the participant circled the same trail multiple times, offering multiple observations of identical positions and 360-degree changes in wind-direction angles.

Data were collected while the participant rode a bicycle in a dynamic outdoor setting. An electric survey vehicle equipped with a suite of environmental sensors followed safely behind the participant during all rides (middle image in Figure 4). Although several dimensions of the environmental context were sampled (e.g., ambient light, temperature, pressure, mass spectra, etc.), here, we focus on the relationship between particulate matter values and biometric variables. Additional relationship will be explored in future works.

Data collection took place in May and June of 2021 at Breckenridge Park located in Richardson, TX over three separate days, which included four to five trials per day. The first two trials consisted of two minutes of eyes closed and eyes open baseline biometric measurements, respectively. The third trial consisted of a “warm-up” ride, where the participant cycled to a public bike trail in tandem with the electric survey vehicle. Additional trials consisted of the participant repeatedly cycling a one-mile loop on a public bike trail. The participant was free to stop cycling at their discretion. Data collection was halted whenever cycling stopped. If the participant chose to continue, a new data collection trial was initiated.

The complete dataset consists of 188 data records collected every 30 s (total time of about 1.5 h) with 329 biometric predictor variables and 51 PM target variables. Biometric predictor variables include: delta (1–3 Hz), theta (4–7 Hz), alpha (8–12 Hz), beta (13–25 Hz), and gamma (25–70 Hz) band power densities for each of the 64 EEG electrodes, body temperature, GSR, HR, HRV, RR, SpO${}_{2}$, average pupil diameter, difference between left and right pupil diameters, and the 3D spatial distance between left and right pupil centers. Environmental PM target variables include: PM${}_{1}$, PM${}_{2.5}$, PM${}_{4}$, PM${}_{10}$, PM${}_{\mathrm{Total}}$, and 45 different PM size bins ranging of 0.18–10 μm measured in μg/m${}^{3}$, as well as particle count density (dCn) measured in P/cm${}^{3}$. The data are publicly available at the Zenodo datastore: https://zenodo.org/record/6326357#.Yieu4RPMJb8, accessed on 29 May 2022 (see Supplementary Materials).

Ethical approval declarations: All experimental protocols were approved by The University of Texas at Dallas Institutional Review Board and informed consent was obtained from the study participant.

### 2.3. Model Development

All models of PM concentration are obtained by an ensemble of decision trees for regression with a hyperparameter optimization process [19,20,21,22,23,24]. Ninety percent of the data is used for training, while 10% is assists as an independent validation dataset. Scripts for model training are freely available at the GitHub repository: https://github.com/mi3nts/DUEDARE, accessed on 29 May 2022 (see Supplementary Materials).

## 3. Results and Discussions

In this work, we used a data-driven experimental paradigm to develop and explore several empirical machine learning models which describe the connection between ambient air particulate matter (PM) concentrations and the biometric variables of an individual breathing that air. Due to logistical constraints imposed by the COVID-19 pandemic, we were only able to collect data from one participant. Additional participants will be included in future research. Two factors, however, mitigate the limited population size in this pilot study. First, the data collection took place over three days, which allowed for contextual variability. Furthermore, the participant repeatedly circled the same trail, allowing for multiple observations of identical spatial positions and 360-degree changes in wind-direction angles.

The estimated PM values included: PM${}_{1}$, PM${}_{2.5}$, PM${}_{4}$, PM${}_{10}$, PM${}_{\mathrm{Total}}$, and 45 different PM size bins ranging of 0.18–10 μm measured in μg/m${}^{3}$, as well as particle count density (dCn) measured in particles per m${}^{3}$. For model development, 329 biometric predictor variables were available. Each machine learning model used was a trained ensemble of decision trees for multi-variate, non-linear, non-parametric regression with full hyperparameter optimization [19,20,21,22,23,24]. The empirical models are evaluated using two key metrics. First, the model accuracy was assessed using the squared correlation coefficient (r${}^{2}$) between the model prediction and the true PM values. Second, a ranking of predictor variable importance was obtained as the weighted average importance of each predictor across the ensemble.

We first evaluated six machine learning models for particulate matter (PM) values, which estimated the particle count density (dCn), PM${}_{1}$, PM${}_{2.5}$, PM${}_{4}$, PM${}_{10}$, and PM${}_{\mathrm{Total}}$. Three hundred and twenty-nine biometric predictor variables were used as model inputs, including delta (1–3 Hz), theta (4–7 Hz), alpha (8–12 Hz), beta (13–25 Hz), and gamma (25–70 Hz) band power densities for each of the 64 EEG electrodes, body temperature, galvanic skin response (GSR), heart rate (HR), heart rate variability (HRV), respiration rate (RR), blood oxygen saturation (SpO${}_{2}$), average pupil diameter, the difference between the left and right pupil diameters (anisocoria), and the 3D spatial distance between the left and right pupil centers (vergence eye movement). Then, using an Occam’s razor principle, the top nine important biometric predictor variables were used to train an additional six models for the same PM variables.

Two subsets of nine biometric predictor variables were used to train two different sets of empirical machine-learning models. The first subset includes EEG variables, and the second subset does not. This first subset was obtained via the Occam’s razor principle mentioned previously, while the second included all nine non-EEG biometric variables from the 329 available biometric predictors. The cognitive effects of air quality can be identified by evaluating predictive models with and without EEG quantities.

**Figure 5:**
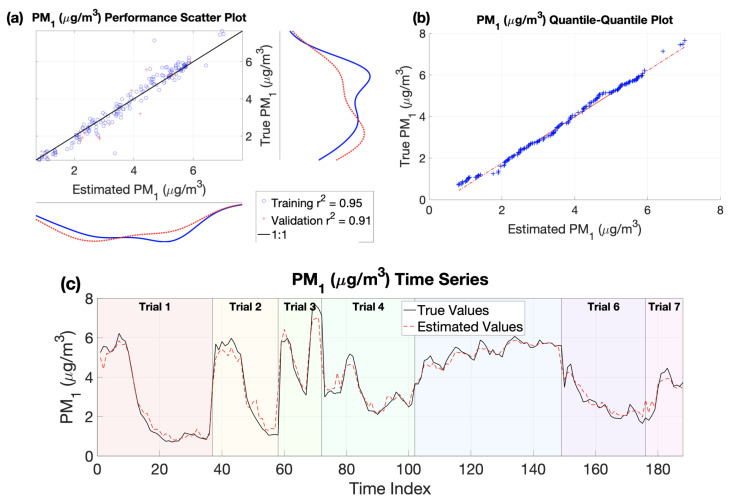
Top performing model (PM${}_{1}$) plots comparing predicted and ground truth values. (a) Scatter plot of true versus predicted PM${}_{1}$ values. A perfect fit is indicated by the 1:1 line shown in black. Training data are shown as blue circles and validation data are plotted as red pluses. (b) Quantile–quantile plot of true versus predict PM${}_{1}$ values. Identical true and predicted distributions would results in a perfect y = x line. (c) Time-series plot of true PM${}_{1}$ values (solid black line) and predicted PM${}_{1}$ values (dashed red line). Background color indicates the trial number associated with each time period. Trials 1–3 were collected on 26 May 2021; trials 4–5 were collected on 9 June 2021; and trials 6–7 were collected on 10 June 2021.

The best performing model using the top nine EEG and non-EEG biometric predictors was for PM${}_{1}$. This model had the highest accuracy with a validation dataset r${}^{2}$ = 0.91. Comparison plots between estimated and ground truth PM${}_{1}$ values are given in Figure 5. In the top-left plot, the estimated and true PM${}_{1}$ concentrations in both the training (blue circles) and validation (red pluses) datasets closely following the perfect fit (black) line. In the top-right plot, the quantile–quantile comparison shows the distribution of measured PM${}_{1}$ values closely resembles the distribution of estimated PM${}_{1}$ values. Finally, in the bottom plot, the time series of the estimated PM${}_{1}$ values (dashed red line) tracks very closely to the true values (solid black line) over seven different trials spanning three separate days.

**Figure 6:**
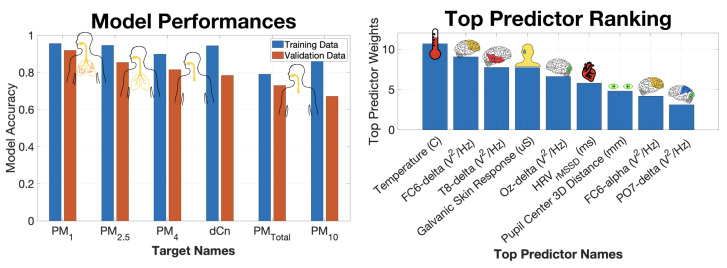
Summary of empirical PM concentration models estimated from 9 EEG and non-EEG biometric predictor variables. (Left) Ranking of model performance defined as squared correlation coefficient between predicted and true PM values. Training and validation dataset performances for each model are shown in blue and orange, respectively. Sorting is based on validation dataset performance. Overlaid graphics indicate the deposition of the respective PM size bins in the airways [25]. (Right) Predictor importance ranking aggregated across all 6 models.

The performances of the PM${}_{1}$ and five other PM models in this cohort are ranked in the left panel of Figure 6. The training and independent validation dataset performances are plotted in blue and orange, respectively, and sorted in descending order of independent validation performance. As previously discussed, PM${}_{1}$ measured in μg/m${}^{3}$ was best reproduced by the nine biometric predictors (validation r${}^{2}$ = 0.91). The empirical models based on the same biometric predictors were less able to accurately estimate the larger PM${}_{10}$ (validation r${}^{2}$ = 0.67) values and PM${}_{\mathrm{Total}}$ (validation r${}^{2}$ = 0.72), which are dominated by PM${}_{10}$ due to the larger masses. The poor performance of these models could be explained by the fact that there are significantly fewer large particles than small particles, and thus the larger particles are not as well mixed as the far more numerous and well-mixed smaller particles. Because of their greater bulk, larger particles settle more quickly. As a result, the concentrations of large particles collected by the survey vehicle and those inhaled by the subject a few meters away are likely to differ more than for the smaller particles. Second, it is possible that the larger particles have less of an impact on the participant’s physical and cognitive state because they are less likely to penetrate deeply into the respiratory and circulatory systems [25].

Each of the six empirical machine learning models has an associated predictor importance ranking, which quantifies the role of individual input predictor variables in estimating the respective PM target variable. The aggregated ranking of top predictors, shown in the right plot in Figure 6, elucidates which biometric variables are most helpful to the empirical models in discerning PM values. The most important predictor variable in estimating PM values was the body temperature measured at the participant’s right temple. Surprisingly, the cardiovascular variable, HRV, played less of a role. Other important biometrics included GSR and the distance between the pupil centers of the eyes. GSR is a strong correlate of body temperature. The distance between the pupil centers is a proxy for vergence eye movements, which have been associated with attentional load and are a strong predictor of cognitive status [26,27]. The delta band (1–3 Hz) power densities for the FC6, T8, and Oz electrodes were found to play an important role in estimating PM values. FC6 is above the frontal cortex on the right side of the head, T8 corresponds to the right temporal lobe, and Oz sits on top of the primary visual cortex.

**Figure 7:**
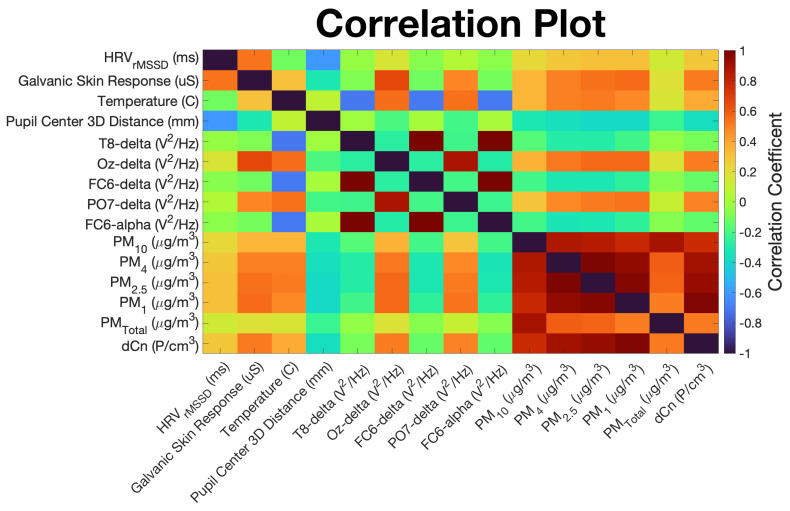
Correlation plot of top 9 EEG and non-EEG biometric predictor variables, along with 6 target PM variables. Positively correlated variable pairs are indicated by a red box, negatively correlated pairs are shown by blue boxes, and non-correlated pairs have green boxes.

Correlations between predictor and target variables are visualized as a color-filled correlation plot in Figure 7. Strong positive correlations are indicated by dark red squares, strong negative correlations are shown by dark blue squares, and the lack of correlation is indicated by green squares. From this plot, we can see HRV, GSR, body temperature, and the delta band power densities of the Oz and PO7 electrode signals have strong positive correlations with all target variables except PM${}_{\mathrm{Total}}$. In other words, as these predictor variables increase, so do the corresponding PM target variables. PM target variables show the greatest negative correlation with the 3D spatial distance between left and right pupil centers, suggesting that the pupils tend to converge with an increase in PM concentrations. Lastly, of all the target variables, PM${}_{\mathrm{Total}}$ is most strongly correlated with PM${}_{10}$ values, which reflects the strong contribution of PM${}_{10}$ particles to PM${}_{\mathrm{Total}}$.

**Figure 8:**
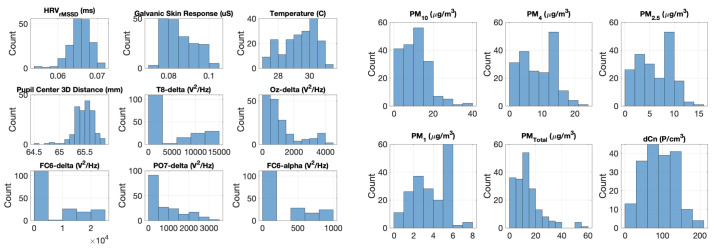
(Left) Histograms of 9 EEG and non-EEG predictor variables. Plots are titled by variable name and its physical units. (Right) Histograms of 6 different PM target variables variables. Plots are titled by variable name and its physical units.

Histograms for both predictor and target variables are displayed in Figure 8. Plots are titled by the variable name and its respective physical units. From the target PM variable histograms in the right plot of Figure 8, the mass scales of different particle sizes are evident. Namely, the larger-sized PM${}_{10}$ particles vary over a much larger range (0–40 μg/m${}^{3}$) than the smaller PM${}_{4}$ (0–20 μg/m${}^{3}$), PM${}_{2.5}$ (0–15 μg/m${}^{3}$), and PM${}_{1}$ particles (0–8 μg/m${}^{3}$). This further explains the strong influence of PM${}_{10}$ values on PM${}_{\mathrm{Total}}$.

Next, an additional set of six empirical machine learning models for the same set of PM targets (dCn, PM${}_{1}$, PM${}_{2.5}$, PM${}_{4}$, PM${}_{10}$, and PM${}_{\mathrm{Total}}$) were evaluated, except this time the PM targets were estimated from nine non-EEG biometric predictor variables (body temperature, GSR, HR, HRV, RR, SpO${}_{2}$, average pupil diameter, difference between left and right pupil diameters, and the 3D spatial distance between left and right pupil centers).

**Figure 9:**
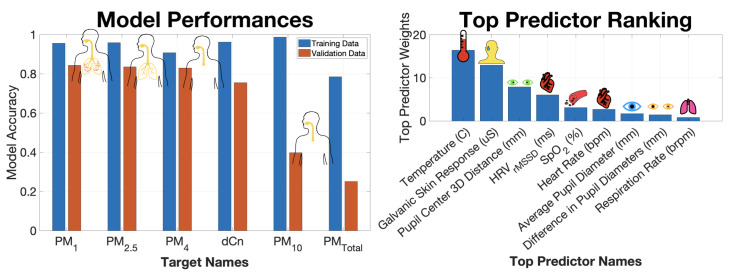
Summary of empirical PM concentration models estimated from 9 non-EEG biometric predictor variables including eye tracking, respiratory, and other physiological variables. (Left) Ranking of model performance defined as squared correlation coefficient between predicted and true PM values. Training and validation dataset performances for each model are shown in blue and orange, respectively. Sorting is based on validation dataset performance. Overlaid graphics indicate the deposition of the respective PM size bins in the airways [25]. (Right) Predictor importance ranking aggregated across all 6 models.

The model performance ranking for the six empirical PM models estimated from the nine non-EEG biometric predictor variables is shown in the left panel of Figure 9. We see that the smaller particles are better estimated by the non-EEG biometrics. Again, this result may be due to better mixing of smaller particles or to deeper penetration of those particles into the respiratory system or both.

Comparing the performance rankings in Figure 6 and Figure 9, there are clear changes in model accuracies. All models with the exception of PM${}_{4}$ exhibit a drop in performance. The largest drop occurs for the already poorly performing PM${}_{\mathrm{Total}}$ (drop in validation r${}^{2}$ = 0.47) and PM${}_{10}$ (drop in validation r${}^{2}$ = 0.28) models.

There is overlap between the importance rankings of Figure 6 and Figure 9. In both cases, body temperature is the most significant predictor of the PM values. Additionally, GSR maintains its order in the ranking as the 2nd most important non-EEG predictors. Although respiratory variables such as HRV and HR appear in the top six of the importance ranking, these variables trail behind temperature, GSR, and the distance between the eye pupil centers.

**Figure 10:**
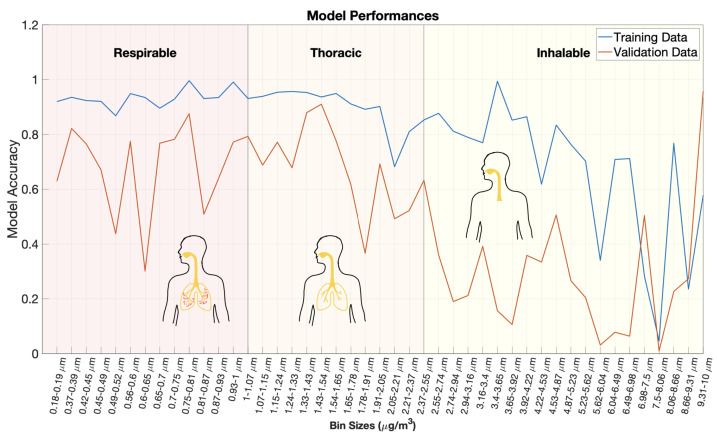
Model accuracies plotted against bin size. Forty-five separate PM models were trained for size bins ranging from 0.18 to 10 micrometers. PM values were estimated solely from 9 non-EEG biometric variables. Training dataset performance is plotted as a blue line and validation dataset performance is plotted in orange. A clear drop in model performance is observed between 2–3 micrometers. Overlaid graphics indicate the deposition of the respective PM size bins in the airways [25,28].

The observation that smaller particles are better estimated than larger-sized particles is explored further by evaluating model performances for a finer particulate size resolution. Here, 45 models were trained to estimate different PM size bins ranging from 0.18 to 10 micrometers using the nine non-EEG biometrics listed above. Model accuracy is plotted against bin size in Figure 10. Training and validation accuracies are plotted as blue and orange lines, respectively. The regional depositions of each particle size bin are indicated by a label and background shading [25,28]. The smallest particles (PM${}_{1}$) are classified as respirable and can penetrate to the alveoli. The next smallest size bin is thoracic (PM${}_{2.5}$) which consists of particle penetrating into the bronchioles. The largest size bin are the inhalable particles (PM${}_{10}$) which can enter into the nose, mouth, and trachea.

There is a clear drop in both training and validation dataset accuracies for size bins between 2 and 3 micrometers, corresponding to thoracic and inhalable particles. For particle size bins above this drop, there is large degree of variation in model performances; however, most have a poor performance with a validation r${}^{2}$ below 0.4. While the results may imply that smaller particles have a greater impact on physiological systems due to their deeper deposition, that conclusion cannot be reached based upon the present data. The drop in performance for larger particles may be explained in part or completely by the fact that smaller particles are more plentiful and better mixed. An evaluation of the relative contributions of each of these factors requires further investigation.

## 4. Conclusions

The human body and its environment form a complex ecosystem. An important aspect of this system is air quality and the impact it has on the human body. Environmental factors trigger physiological responses that can be detected by holistic biometric sensing. In this prototype study, we used an ultra-fine holistic sensing paradigm to demonstrate how particulate matter concentrations in the ambient environment can be accurately estimated using only nine biometric variables. In addition, smaller particles were found to be more accurately estimated. Two potential causes may explain this result. First, smaller particles are much more abundant and well mixed in the ambient environment than larger ones, thus resulting in a greater similarity between particles inhaled by the participant and collected by the survey vehicle. Secondly, smaller particles can deposit into the respiratory system more deeply, and may have a greater impact on the body. Further investigation is needed to assess the relative contributions, if any, of these two factors, since they are not mutually exclusive.

The largest limitation of this work is that data collection was restricted to a single participant. While it is not clear if the observations from this pilot study will extend to a broader population, we have laid a foundation for future investigations of environmental impacts on human physiology on ultra-fine scales. Future research will include data from multiple participants. Additionally, several other environmental variables were collected (e.g., ambient light, temperature, black carbon, ozone, NO/NO${}_{2}$/NO${}_{x}$, etc.) and will be evaluated for their physiological interactions. By understanding the key interactions between the environment and the human body, health and performance can be improved across a variety of domains.

## Data Availability

The data used in this study are publicly available at the Zenodo datastore: https://zenodo.org/record/6326357#.Yieu4RPMJb8, accessed on 29 May 2022.

## Supplementary Materials

The data and code have been made publicly available. The full data set is available at the Zenodo data store: https://zenodo.org/record/6326357#.Yieu4RPMJb8 (accessed on 29 May 2022) and code is available at the GitHub: https://github.com/mi3nts/DUEDARE (accessed on 29 May 2022).

## Abbreviations

The following abbreviations are used in this manuscript:

WHO	World Health Organization
HR	Heart Rate
BP	Blood Pressure
EEG	Electroencephalography
GSR	Galvanic Skin Response
ECG	Electrocardiography
HRV	Heart Rate Variability
PPG	Photoplethysmogram
RR	Respiration Rate
PM	Particulate Matter
dCn	Total Particle Count Density
SpO${}_{2}$	Blood Oxygen Saturation
